# Energetically expensive dynamo action in Earth’s basal magma ocean

**DOI:** 10.1073/pnas.2507575122

**Published:** 2025-11-03

**Authors:** Nathanaël Schaeffer, Stéphane Labrosse, Jonathan M. Aurnou

**Affiliations:** ^a^Université Grenoble Alpes, Université Savoie Mont Blanc, Université Gustave Eiffel, Institut des Sciences de la Terre, Grenoble 38000, France; ^b^Laboratoire de géologie de Lyon: Terre, Planètes, Environnements, École Normale Supérieure de Lyon, Lyon, 69003, France; ^c^Department of Earth, Planetary, and Space Sciences, University of California, Los Angeles, CA 90095-1567

**Keywords:** geomagnetism, dynamo, early Earth, magma ocean, convection

## Abstract

Previous studies focusing on the electrical conductivity and thermal evolution of an early magma ocean at the base of Earth’s mantle have found that basal magma ocean (BMO) convection could have produced the ancient geomagnetic field. By advances in high-resolution dynamo modeling, we find that convection in a thin BMO-like spherical shell is able to sustain strong magnetic fields, including axial dipolar fields similar to current day field structure. However, integrating our dynamo results with improved thermal evolution models and taking the planet’s rapid rotation into account using rotating convective turbulence models implies that an Earth-like magnetic field was unlikely to have been generated in the BMO, a finding relevant to the interpretation of ancient paleomagnetic signatures, Earth’s global-scale dynamics, and long-term planetary evolution. Large uncertainties still remain, calling for refined models of deep Earth thermal and mineralogical processes, accurate determination of prefactors in convective scaling laws, and fully coupled core-BMO dynamo simulations. Nonetheless, our work highlights that BMO-type dynamos intrinsically require a larger product of electrical conductivity and velocity than core-type dynamos, and that they are similarly rotationally constrained, so that velocities are significantly reduced compared to nonrotating estimates.

## Significance

It is argued that the early geomagnetic field must have been maintained in a relatively thin molten silicate ocean situated just above Earth’s liquid metal core. Our detailed 3D numerical models show that such thin layer dynamos can indeed produce strong dipolar fields. Their generation, however, requires the electrical conductivity of the basal magma ocean (BMO) fluid to be roughly a factor of five larger than current estimates. Another mechanism may thus be required to explain Earth’s ancient magnetic field. Numerous open questions still persist, highlighting the need for further research into the deep Earth geophysical processes involved and their specific impacts on BMO dynamo. Nonetheless, achieving dynamo action in a BMO is certainly more challenging than previously surmised.

Earth’s magma ocean solidified last at the bottom of the mantle, thus forming a basal magma ocean (BMO) (1, 2). The solidification of the BMO likely released so much latent heat that it would have limited the heat that emanated from Earth’s molten metal core. In this scenario, the iron-rich core then becomes stably stratified and thermally driven convection does not occur in the core. Early generation of the geomagnetic field (3, 4) would then have had to occur elsewhere within the planet.

The most likely candidate for hosting a long-lived dynamo outside the core is within the BMO itself (5–8). Ab initio calculations found that the electrical conductivity σ of the BMO fluid could have been relatively high, $\sigma \approx 2\times 10^{4}$ S/m, roughly 1% that of core fluid (9, 10). Employing these σ values allowed (6) to argue that BMO dynamo action could have occurred for at least the first 2 billion years of Earth’s existence. To reach this conclusion, the magnetic Reynolds number Rm∝σUL, was calculated based on molten silicate σ values along with estimates of the BMO convective velocity scale U and fluid layer thickness L. Importantly, U was calculated using nonmagnetic, nonrotating mixing length relationships and the thermal buoyancy flux estimates of ref. 1. The critical magnetic Reynolds number, $Rm_{\text{crit}}$, above which dynamo action is sustainable, was taken to be 40, based on Earth’s core dynamo models that use a spherical shell inner-to-outer radius ratio value of χ=0.35 (11), a value far thicker than applies to any potential BMO. It was further assumed that strong zonal shearing flows in the full volume of Earth’s stably stratified core would generate strong toroidal magnetic fields that would act to bolster the dynamo action occurring within the convecting BMO (cf., ref. 12).

Here we carry out high resolution, thin shell dynamo models, providing direct estimates of $Rm_{\text{crit}}$ in a BMO-like system. In addition, i) we update the BMO convection physics, using the well-established rotating convection velocities found in theory, numerical simulations and laboratory experiments (e.g., refs. 13–21) and ii) we update the thermal evolution model of Labrosse et al. (1), primarily to account for more realistic phase diagrams and crystallization-induced compositional buoyancy fluxes during BMO solidification. Altogether, these allow us to test the essential ideas underlying the early Earth BMO dynamo hypothesis. The majority of our BMO dynamo models are carried out in a $\chi =r_{i}/r_{o}=0.9$ radius ratio shell, corresponding to a BMO radial depth extent of L=390 km. A smaller number of χ=0.8 calculations are made, corresponding to deeper, L=870 km thick BMO.

Stress-free mechanical boundaries minimize viscous boundary drag, because the viscosity we can afford numerically is at least 6 orders of magnitude higher than that of the magma ocean. The buoyancy forcing is set to drive rapidly rotating turbulent convection. The most demanding models are eightfold symmetric in azimuth, $m_{\mathrm{fold}}=8$, in order to make tractable the highest resolution cases. The buoyancy flux is set to zero on the core–mantle boundary (on the spherical fluid shell’s inner radius $r=r_{i}$) and to a nonzero value on the outer radius atop the BMO ($r=r_{o}$). Although the boundary conditions on the buoyancy field are not fully realistic, it has been shown that they have little influence on turbulent rotating convective flows (e.g., refs. 22 and 23). However, an influence on the magnetic field generation still remains possible, as this has not been formally investigated to date. The metallic core ($r<r_{i}$) is treated as an electrically insulating solid. This choice allows us to reach turbulent flow regimes relevant for magma oceans at the expense of neglecting the influence of the assumed stably stratified core. In particular, zonal flows in the core are not modeled here. This simplification is made in light of ref. 12, in which it was shown that boundary-forced zonal flows do not penetrate a stably stratified core, and therefore do not generate strong magnetic fields. Further, insulating vs. conducting solid inner core models have been shown to only negligibly influence the field structure in geodynamo simulations (24). These simplifying assumptions will be lifted in future models that investigate the effects of an electrically conducting, stably stratified fluid outer core on BMO dynamo action. See *Materials and Methods* for details of the governing equations and the XSHELLS dynamo code employed (25, 26).

Dynamo action is sustained by convective turbulence in planets and stars. Thus, in each set of dynamo simulations, we fix the value of the heat flux (Rayleigh number, $Ra_{F}$) and the fluid viscosity (Ekman number, E), and vary the fluid’s electrical conductivity, σ (corresponding nondimensionally to the magnetic Prandtl number, Pm), with all other control parameters held fixed throughout. The buoyancy flux, $Ra_{F}$, at a given fluid viscosity is set such that the convective flow is turbulent at all latitudes (27), while keeping a strong influence of the planet’s spin on the flow (Rossby number Ro=ReE≪1).

**Fig. 1.**
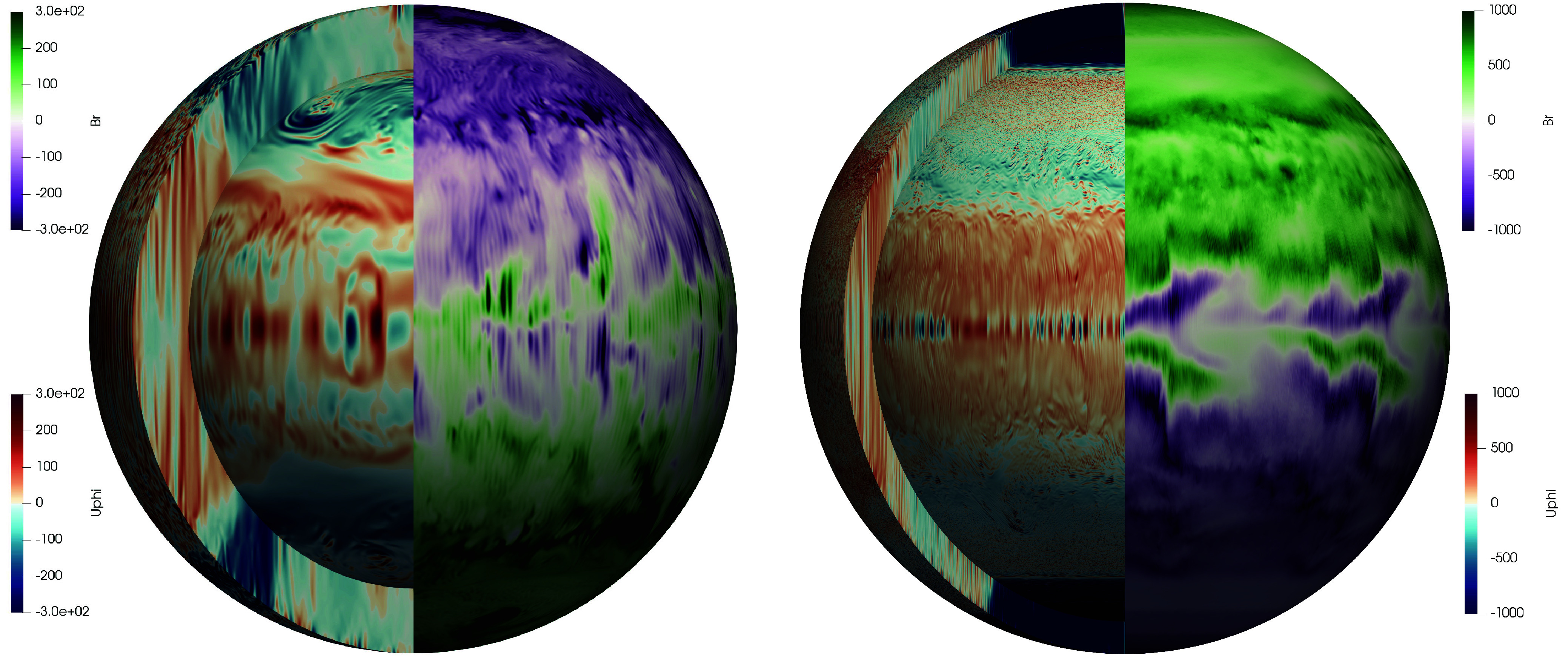
Turbulent thin-shelled BMO dynamo simulations. (*Left*) deeper $\chi =r_{i}/r_{o}=0.8$ model with $E=10^{-5}$, $Ra_{F}=2.5\times 10^{9}$, Pm=1, Pr=5 and no imposed azimuthal symmetry ($m_{\mathrm{fold}}=1$). (*Right*) shallower χ=0.9 model with $E=10^{-6}$, $Ra_{F}=10^{11}$, Pm=0.25, Pr=5 model and imposed eightfold azimuthal symmetry ($m_{\mathrm{fold}}=8$). The snapshots show the azimuthal velocity, $U_{\phi }$, on the outer radius $r_{o}$, the inner radius $r_{i}$ and in a meridional slice (left hemisphere), alongside the dominantly axially aligned, dipolar radial magnetic field, $B_{r}$, shown on the BMO’s outer surface $r_{o}$ (right hemisphere). Velocity is given in Reynolds number units, $Re=U_{\phi }L/\nu$, where $L=r_{o}-r_{i}$ is the BMO shell thickness and ν is the fluid’s kinematic viscosity. Magnetic field strength is presented here in Alfvén velocity units $V_{A}L/\nu =B_{r}L/\left(\sqrt{\rho \mu_{o}}\nu \right)$, where ρ is fluid density and $\mu_{o}$ is magnetic permittivity.

Fig. 1 shows 3D views from two of our BMO dynamo simulations. The left-hand image is a snapshot from a slightly thicker shelled χ=0.8, $E=10^{-5}$ case made with no imposed azimuthal symmetry ($m_{\mathrm{fold}}=1$) and $Ra_{F}=2.5\times 10^{9}$, Pm=1 and Pr=5, with nondimensional parameter definitions given in *Materials and Methods*. The right-hand snapshot is from one of the most turbulent (Rm=137; Re=550), lowest viscosity ($E=10^{-6}$), thin shelled (χ=0.9) convective BMO dynamo cases ($Ra_{F}=10^{11}$, Pm=0.25, Pr=5, $m_{\mathrm{fold}}=8$). The azimuthal velocity field is rendered on the left hand side of each image. The importance of rotation is evinced by the nearly columnar convection-scale flow structures (e.g., refs. 26 and 28). Except for a pair of strongly anticyclonic polar vortices in the $E=10^{-6}$ case, strong zonal flows are kept in check via electromagnetic breaking by the dynamo-generated magnetic field (e.g., refs. 29–31). The right hand side of each image displays the radial magnetic field on the model’s outer boundary $r_{o}$. The dynamo magnetic fields are predominantly dipolar. Further, the magnetic energy is contained mainly in the axial component of the dipole field, a byproduct of the rotationally constrained dynamics that is also bolstered by the eight-fold azimuthal symmetry in the $E=10^{-6}$ case.

Starting from high σ and moving to successively lower values, it is possible to determine the minimum, or “critical,” electrical conductivity $\sigma_{\text{crit}}$ needed for a given setup to generate dynamo magnetic fields. Since Pm∝σ, this method identifies $Pm_{\text{crit}}$ as well as the critical value of the magnetic Reynolds number, $Rm_{\text{crit}}$, which denotes the ratio of magnetic induction to dissipation. It must be noted that Rm=RePm∝σU is sensitive to the value of σ and the characteristic fluid velocity U, which is also sensitive to σ (e.g., refs. 32 and 33). Thus, $\sigma_{\text{crit}}$ and $Rm_{\text{crit}}$ both denote the limit of dynamo action, but do not linearly covary in our fully dynamic simulations. Table 1 contains estimates of the lowest magnetic Prandtl and Reynolds numbers, $Pm_{\text{crit}}$ and $Rm_{\text{crit}}$, at which thin-shell dynamo action is found for each of the four fluid viscosities investigated, $E=10^{-3}$, $10^{-4}$, $10^{-5}$, and $10^{-6}$. By reducing the fluid viscosity toward BMO-relevant values ($E∼10^{-12}$; see *Materials and Methods*), we aim to approach the asymptotic regime. The four Ekman numbers investigated at χ=0.9 yield numerically derived $Rm_{\text{crit}}$ values that range between approximately 70 and 170. Our thinner shell simulations show that $Rm_{\text{crit}}\left(\chi =0.9\right)≳2Rm_{\text{crit}}\left(\chi =0.35\right)$. Thus, the onset of dynamo action requires Rm values that are at least twice the value necessary to generate dynamo action in thick shelled χ=0.35 outer core dynamo simulations.

**Table 1.** Critical magnetic Prandtl and Reynolds number values, *Pm*_crit_ and *Rm*_crit_ below which dynamo action does not occur and the minimum magnetic Reynolds number for *ME/KE* > 1 strong-field dynamo action *Rm*_strong_, as a function of nondimensional fluid viscosity *E* and radius ratio *χ* = *r_i_*/*r_o_*.

E	χ	$Pm_{\text{crit}}$	$Rm_{\text{crit}}$	$Rm_{\text{strong}}$
$10^{-3}$	0.9	1.45±0.05	165±1	—
$10^{-4}$	0.9	0.4±0.1	164±16	—
$10^{-5}$	0.8	0.19±0.01	62±4	≈135
$10^{-5}$	0.9	0.14±0.01	71±6	≈85
$10^{-6}$	0.9	0.06±0.01	≈80	≈130

The characteristic convective velocity Re can be estimated from Rm=RePm.

Due to the significant computational cost associated with simulating turbulence in thin shell geometries (cf., ref. 34), achieving more realistic values remains challenging with current supercomputing resources. Importantly, the amplitudes of the terms in our equations are appropriately ordered in all our $E\leq 10^{-5}$ simulations, indicating that the overall system dynamics are likely well captured (35). While some fluctuation in the value of $Rm_{\text{crit}}$ is possible while traveling to more realistic conditions, we expect that the $Rm_{\text{crit}}$ ordering presented here is robust and will not vary substantially (such that $Rm_{\mathrm{crit}}\left(\chi =0.9\right)>Rm_{\mathrm{crit}}\left(\chi =0.35\right)$ as E→0). In basic support of this contention, the χ=0.9 simulations at the two lowest viscosities, $E=10^{-5},\;10^{-6}$, are consistent with $Rm_{\text{crit}}∼75\pm 5$. Further, we find that χ=0.8 dynamo action onsets at $Rm_{\text{crit}}\approx 60$, a value which, somewhat reassuringly, lies between the $Rm_{\text{crit}}\left(\chi =0.35\right)\approx 40$ core dynamo findings (36) and our $Rm_{\text{crit}}\left(\chi =0.9\right)\approx 70$ to 80 results.

Our extreme BMO dynamo simulations demonstrate that rapidly rotating, space-filling turbulence in a geometrically thin shell can generate large-scale axial dipolar magnetic fields, broadly similar in structure to that of the current-day geomagnetic field (Fig. 1). However, these models require more vigorous convection ($Rm_{\text{crit}}≳70$) to generate dynamo action compared to prior thick shelled models ($Rm_{\text{crit}}\approx 40$). This increase in $Rm_{\text{crit}}$ is geophysically important in that it removes the possibility of early dynamo action in the thermal evolution models explored below.

**Fig. 2.**
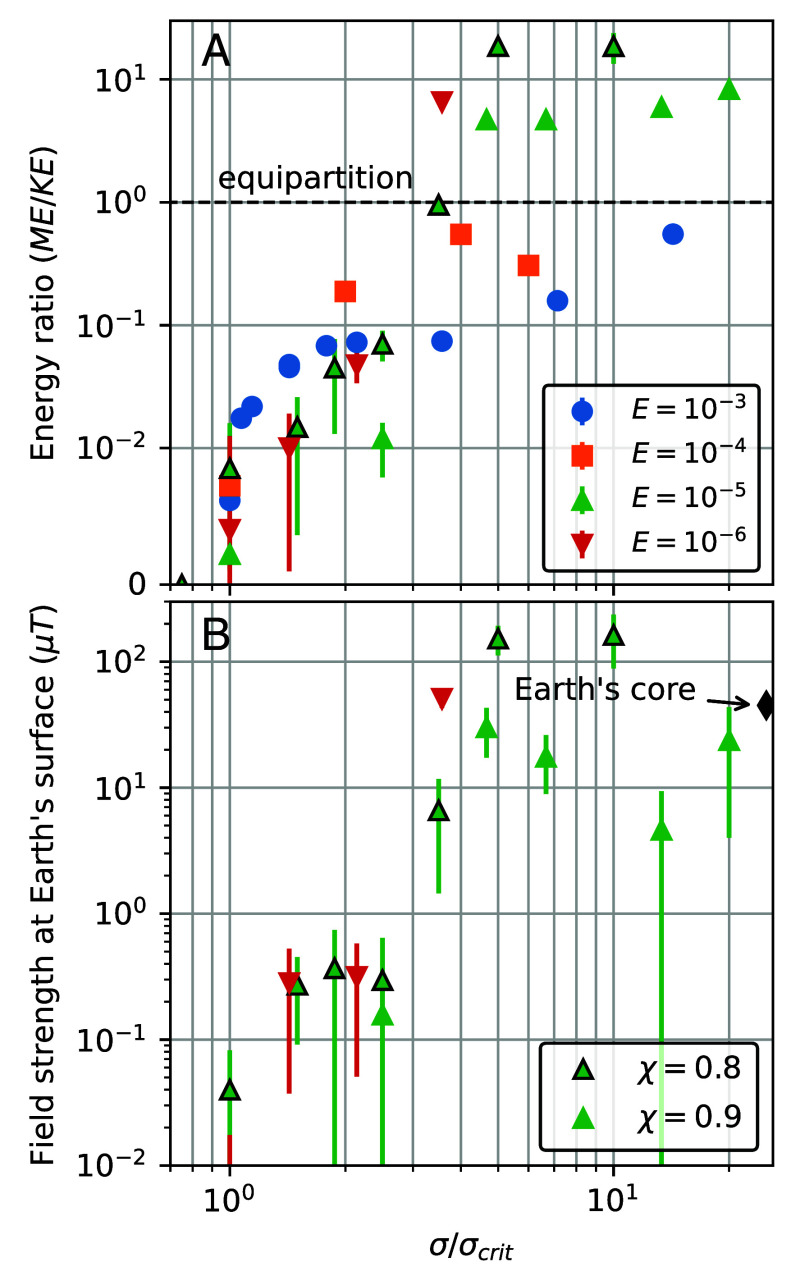
Magnetic field intensities in BMO dynamo simulations as a function of the normalized electric conductivity, $\sigma /\sigma_{\text{crit}}$, for the four fluid viscosities E and the two radius ratios investigated. (*A*) Ratio of volume-integrated magnetic, ME, and kinetic energies, KE. Equipartition, where ME/KE=1, is marked by the horizontal black dashed line. (*B*) Simulated dipole strength at Earth’s surface, converted from an Elsasser number $\Lambda_{\mathrm{dipole}}=\sigma B{\left(r_{o}\right)}_{\mathrm{dipole}}^{2}/\left(2\rho \Omega \right)$ measured at the dynamo surface $r_{o}$, and extrapolated to Earth’s surface, where the current core field gives 45 μT. Only $E\leq 10^{-5}$ cases are shown since only they feature strong-field dynamos. Symbols outlined in black denote χ=0.8 cases; all others are χ=0.9.

In addition to magnetic field morphology and existence, we also investigate the magnetic field intensity. Close to dynamo onset, at $Rm/Rm_{\text{crit}}∼\sigma /\sigma_{\text{crit}}≃1$, the volume-integrated energy contained in the magnetic field, ME, is always weak compared to the kinetic energy of the fluid motions, KE. This is shown in Fig. 2*A*, where ME/KE values are plotted vs. the electrical conductivity ratio $\sigma /\sigma_{\text{crit}}$. Near $\sigma /\sigma_{\text{crit}}≃1$, the energy ratio is far less than unity, whereas this ratio currently exceeds $10^{3}$ in Earth’s core (26, 37). Thus, these $\sigma \approx \sigma_{\text{crit}}$ cases are considered “weak field” dynamo solutions (e.g., ref. 38). Extrapolating these solutions to Earth’s surface yields surface dipolar magnetic fields that are much smaller than the current geomagnetic field, as shown in Fig. 2*B*. With increasing $\sigma /\sigma_{\text{crit}}$, the conversion from kinetic energy to magnetic energy becomes more efficient. The Earth-like, ME>KE “strong field” regime requires significantly stronger turbulent induction, corresponding to $Rm\approx 85\;\mathrm{to}\;130≃1.2\;\mathrm{to}\;2Rm_{\text{crit}}$ and $\sigma ≳3\sigma_{\text{crit}}$ (Table 1 and Figs. 2 and 6). In the strong field regime, the planetary surface dipole magnetic field can reach amplitudes within the range of paleointensities (4, 39), or within an order of magnitude of the current geomagnetic field. Our turbulent BMO dynamos also challenge the prevailing view that thin shell dynamos cannot produce strong axial dipolar fields under realistic (E≪1; Ro≪1; Pm<1) conditions (cf., refs. 40–42).

With these thin shell dynamo modeling results in hand, we turn to thermal evolution modeling to best characterize the BMO’s dynamo-generation state through Earth’s history. Labrosse et al. (1) made the first models of BMO evolution, which were then adapted to test the plausibility of BMO dynamo action (5, 6). Here, we follow the same modeling approach with improvements regarding the phase diagram (2) and the compositional energy calculation (43). In addition, the buoyancy flux to drive convection in the BMO is computed differently from previous studies: i) the latent heat released at the top of the BMO is subtracted from the total heat flux to compute the thermal buoyancy and ii) the compositional buoyancy is included due to FeO release upon fractional crystallization of MgO-rich solid at the BMO’s outer boundary (44, 45).

**Fig. 3.**
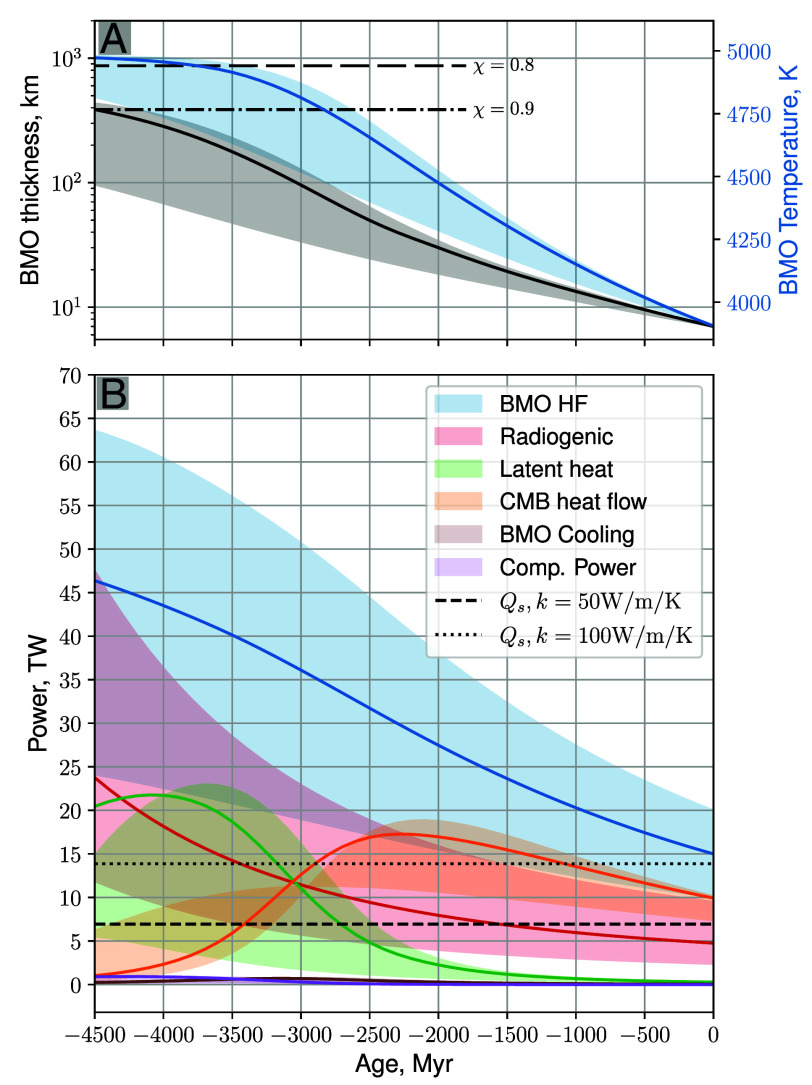
Thermal evolution modeling for current outward BMO heat flows of $Q_{0}=10,15$, and 20 TW. The $Q_{0}=15\;\text{T W}$ evolutionary path for each quantity is plotted as solid line, the shaded area giving the range between the $Q_{0}=10$ and 20 TW scenarios. (*A*) BMO thickness $L=r_{o}-r_{i}$ (gray and black, left-hand ordinate) and bulk temperature (blue, right-hand ordinate). The horizontal dashed and dash-dotted lines give the thickness values corresponding to $\chi =r_{i}/r_{o}=0.8$ and 0.9, respectively. (*B*) Curves showing the heat flux leaving the BMO into the overlying mantle (blue); radiogenic BMO heating (red); latent heat of crystallization at $r_{o}$ (green); CMB heat flow (orange); BMO secular cooling (brown); BMO compositional power (purple), all in units of TW. The horizontal dashed and dotted lines denote the isentropic CMB heat flow at the top of the core for core thermal conductivities $k_{c}=50$ and 100 mW m^−1^ K^−1^, respectively.

**Fig. 4.**
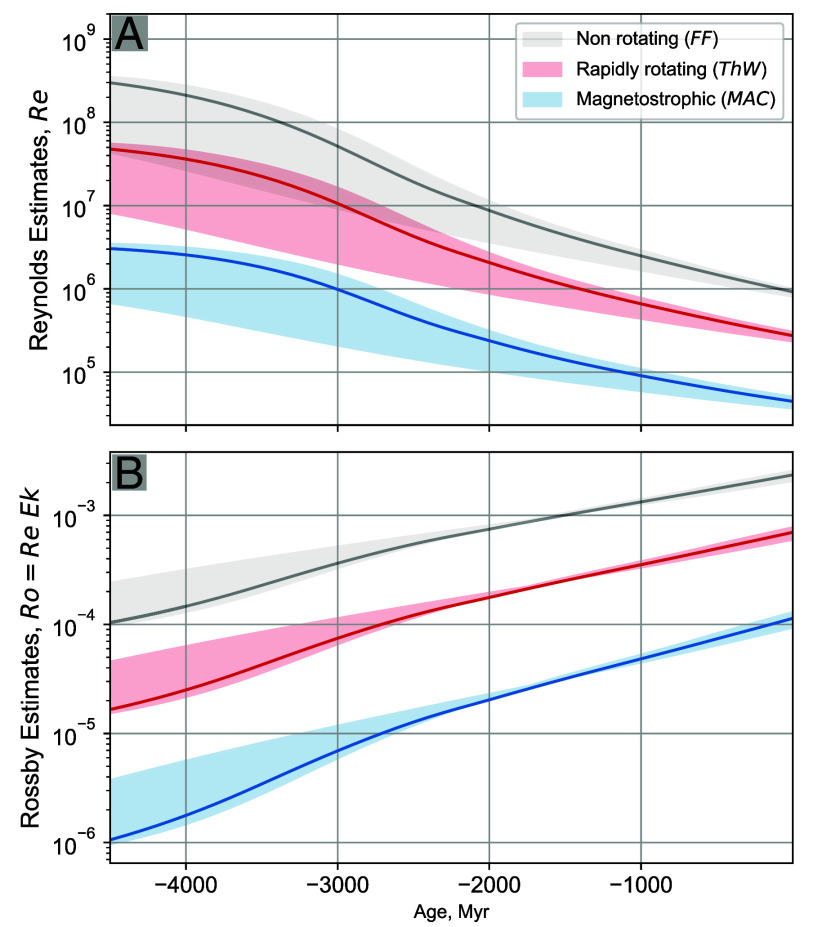
Nondimensional BMO convective velocity estimates based on the power outputs from thermal evolution model for current heat flow $Q_{0}=$10, 15 and 20 TW. (*A*) Reynolds numbers, Re, estimate the ratio of inertial to viscous forces, whereas (*B*) Rossby numbers, Ro=ReE, measure the ratio of inertial to Coriolis forces. The gray curve corresponds to the nonrotating free-fall (FF) velocity scaling. The red curve marks the rapidly rotating thermal wind (ThW) scaling. The blue curve denotes the magnetostrophic (MAC) scaling.

We consider evolution models in which the current-day outward heat flux takes three different values, $Q_{0}=10,\;15$, and 20 TW, with total radiogenic heating in the BMO accounting respectively for $f_{\mathrm{rad}}=1/8$, 1/4, and 1/2 of the total budget of the bulk silicate Earth, 20 TW. Jointly varying the heat flow at the bottom of the solid mantle and the radiogenic heating in the BMO is justified by the fact that total heat flow at the surface of the Earth is well established (46) and provides a loose constraint on the sum of these two terms. The range of $Q_{0}$ and $f_{\mathrm{rad}}$ values used here are reasonable (1, 47–49) and allow us to investigate the effect of varying the energy balance of the BMO on its potential for dynamo action. The model also accounts for the decrease in planetary rotation rate over geologic time, here following ref. 50. Our thermal evolution modeling results are presented in Figs. 2–4, each quantity being represented as a shaded region extending between the $Q_{0}=10$ to 20 TW paths, along with the $Q_{0}=15$ TW pathway shown as a solid line. The thermal evolution equations are given in *Materials and Methods*.

Fig. 3*A* shows BMO thickness $L=r_{o}-r_{i}$ in black and BMO bulk temperature in Kelvin in blue from the thermal evolution model with various values of the current outward BMO heat flux out $Q_{0}$. Integrating backward through time (denoted here as “age”), this model yields an initial BMO thickness in the range L≃95 to 500 km, corresponding to an initial spherical shell radius ratio in the range χ=0.87 to 0.97. Using an alternative phase diagram with an even stronger Fe fractionation during crystallization yields a larger initial BMO thickness, L=780km at most (*Materials and Methods* and *SI Appendix*), corresponding to χ=0.81. Our dynamo models are all carried out using χ=0.9 and 0.8 (L≃390 and 870 km), which falls in the range of values obtained in the early times predicted by our models.

Fig. 3*B* shows the power available to sustain BMO convective flows in our thermal evolution models. The total heat flow from the BMO into the overlying solid mantle is given by the blue curve. The red curve is the radiogenic heating, $H_{\mathrm{rad}}$, within the BMO. The latent heat of freezing is shown by the green curve and the orange curve describes the heat flow out from the core. The buoyant power released by compositional evolution of the BMO (purple) and BMO secular cooling (brown) are small contributors to the total budget. Note that the CMB heat flow is lower than the isentropic value, as represented by dashed and dotted lines for a core thermal conductivity of $k_{c}=50$ and 100 mW m^−1^ K^−1^, for the first ∼1 to 2 Gyr of planetary evolution. In that period, the core fluid is expected to be stably stratified in the absence of a growing solid inner core, justifying the need for an alternative way to sustain the magnetic field.

This updated thermal evolution model provides more accurate estimates of the mixed thermocompositional buoyancy available to drive rotating convective BMO turbulence. We use the results in Fig. 3*B* to provide estimates of characteristic velocities in the convecting BMO, using the velocity scalings (14, 16, 18, 21) presented in *Materials and Methods*. These velocity estimates are shown in Fig. 4*A* in terms of nondimensional Reynolds number values. The nonrotating mixing length velocity corresponds to the free-fall (FF) limit (51) and is shown by the gray curve. In rapidly rotating systems, the limiting “thermal wind” velocity (ThW; red curve) is set by the balance between available buoyancy and axial vortex stretching (13, 14, 16–21, 52). For strong magnetic fields, the magnetostrophic velocity (MAC; blue curve) is predicted to emerge, arising from a balance between buoyancy, Coriolis, and Lorentz forces (14, 36, 38, 53–57). The nonrotating free-fall velocities have the highest predicted amplitudes, with the rotating thermal wind velocities roughly an order of magnitude smaller and the magnetostrophic velocities more than two orders of magnitude smaller than the FF values.

Fig. 4*B* shows estimates of the Rossby number, Ro=ReE, which estimates the ratio of the inertial and Coriolis accelerations in the fluid layer. When Ro≫1, inertial effects dominate rotation and convective flows are essentially nonrotating; when Ro≪1, the flows exist in the rapidly rotating regime (16). All the Ro values are well below unity in Fig. 4*B*. Importantly, this implies that the nonrotating FF velocities cannot be used to describe the Ro≪1 flows in the convecting BMO (cf., ref. 6), or in any other rapidly rotating Ro≪1 fluid layers (cf., refs. 58 and 59). Only the ThW and MAC branches may apply, as both are possible when Ro≪1.

**Fig. 5.**
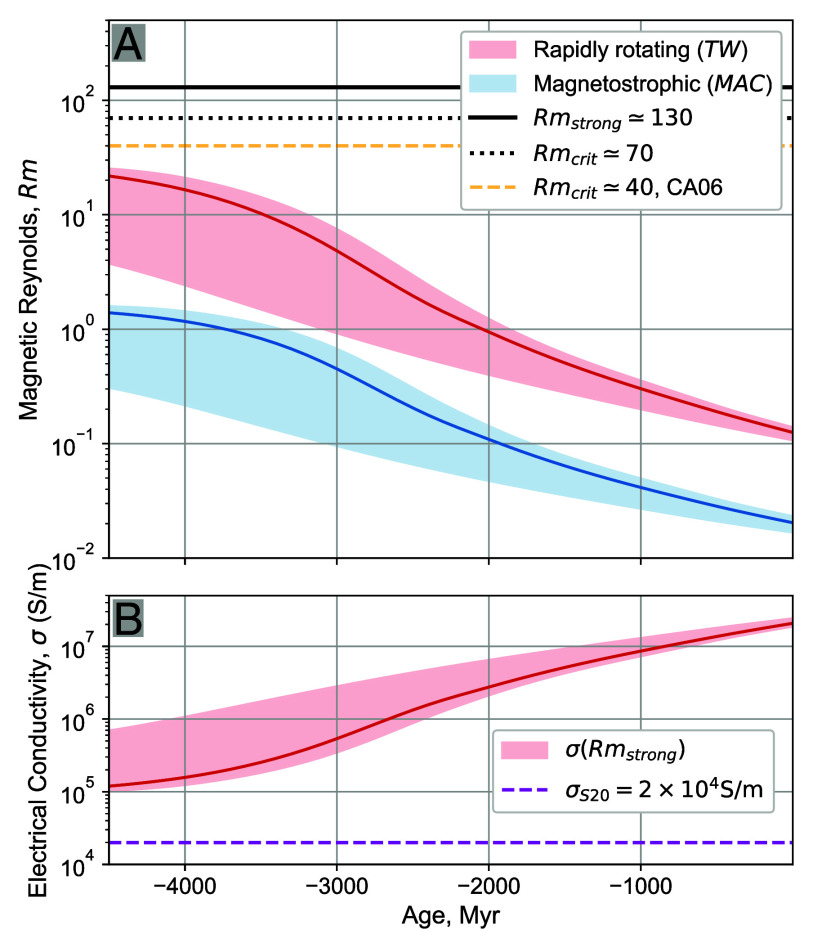
(*A*) Magnetic Reynolds number Rm as a function of time for the different velocity scalings considered here. The critical value, $Rm_{\text{crit}}$, above which dynamo action occurs is estimated to be ≈70 in our thin shell BMO dynamo models (black dashed line) and ≈40 in thick shell outer core models (orange dashed line). (*B*) Estimated BMO electrical conductivities, $\sigma \approx Rm_{\text{strong}}/\left(\mu_{o}U_{\mathrm{ThW}}L\right)$, necessary for $\chi =0.9,\;E=10^{-6}$ strong field dynamo action driven via thermal wind flows on the thermal evolutionary paths modeled here. For comparison, the magenta dot-dashed line denotes $\sigma_{S20}=2\times 10^{4}$ S/m from ref. 6.

Fig. 5*A* shows the temporal variation of the magnetic Reynolds number, calculated as $Rm=RePm=\mu_{o}\sigma UL$, where $\mu_{o}$ is the magnetic permittivity of free space, $\sigma =2\times 10^{4}$ S/m is taken from Stixrude et al. (6), and U is the characteristic convective velocity. The red and blue curves use the rapidly rotating ThW and MAC velocity estimates, respectively. The dashed orange horizontal line shows the estimated value, $Rm_{\text{crit}}\approx 40$, from thick shelled outer core dynamo models (11). The dashed black line denotes the lowest critical magnetic Reynolds number, $Rm_{\text{crit}}\approx 70$, for dynamo action in our BMO simulations. The solid black line corresponds to the magnetic Reynolds number, $Rm_{\text{strong}}\approx 130$, at which strong field BMO dynamo action is found in our lowest viscosity $E=10^{-6}$ cases.

Fig. 5*A* does not show the velocity estimate based on the nonrotating *FF* scaling. This is the case because nonrotating dynamics (e.g., refs. 13, 16, 21, and 60) neither characterize nor occur in Ro ≪1 rapidly rotating convection systems (e.g., refs. 15–20, 29, 36, and 61–63). A figure using the FF scaling in *SI Appendix* suggests that a strong-field BMO dynamo, with $Rm≳Rm_{\text{strong}}$, could have existed for ≲0.5 Gyr, in basic agreement with Stixrude et al. (6). However, the free-fall velocity scaling only holds in slowly rotating systems in which the Rossby number Ro is unity or greater (16). Per Fig. 4*B*, it is clear that Ro≪1 for all three velocity scalings over Earth’s entire history. Thus, the FF scaling does not pertain to convection within Earth’s Ro≪1 BMO. If instead MAC estimates are employed then the BMO magnetic Reynolds numbers never exceed 2; BMO dynamo action cannot occur in the magnetostrophic regime. This leaves rapidly rotating thermal wind velocities to provide the only potential dynamo generating BMO flows.

For the range of heat flows considered in our thermal evolution models, thermal wind flows generate Rm<30. Thus, the Rm curves never exceed our thin shell $Rm_{\text{crit}}\approx 60$ to 80 values, but they nearly reach the χ=0.35 outer core thick shell $Rm_{\text{crit}}\approx 40$ value during the first 0.5 Gyr of Earth’s evolution. Only slightly larger Rm values are obtained by using a phase diagram with a larger amount of Fe fractionation (*SI Appendix*), such that BMO dynamo action would still not be possible along these thermal evolutionary paths.

Fig. 5*B* shows the critical value of the BMO electrical conductivity for strong field dynamo action to arise. Here we have used the thin shell χ=0.9, low viscosity $E=10^{-6}$ estimate of $Rm_{\text{strong}}=130$. Inverting the Rm definition then yields $\sigma_{\text{strong}}\approx Rm_{\text{strong}}/\left(\mu_{o}UL\right)$. Thermal wind velocities are used for U and BMO layer depths L are taken from thermal evolution models with the same range of heat flow as before. Although neither $\sigma_{\text{strong}}$ curve intersects the Stixrude et al. (6) value, $\sigma_{S20}$ (magenta, dashed line), they are not far removed. The BMO’s electrical conductivity could evolve over time, parallel to its FeO content (Fig. 7 in *Materials and Methods*). If a value of $10\sigma_{S20}$ is reached early in Earth’s history, strong-field BMO dynamo action could be viable along the 20 TW pathway for the first 1 Gyr of Earth’s evolution. Higher conductivities of order $50\sigma_{S20}$ are necessary for the early existence of $Q_{0}=10$ TW strong field dynamo action.

**Fig. 6.**
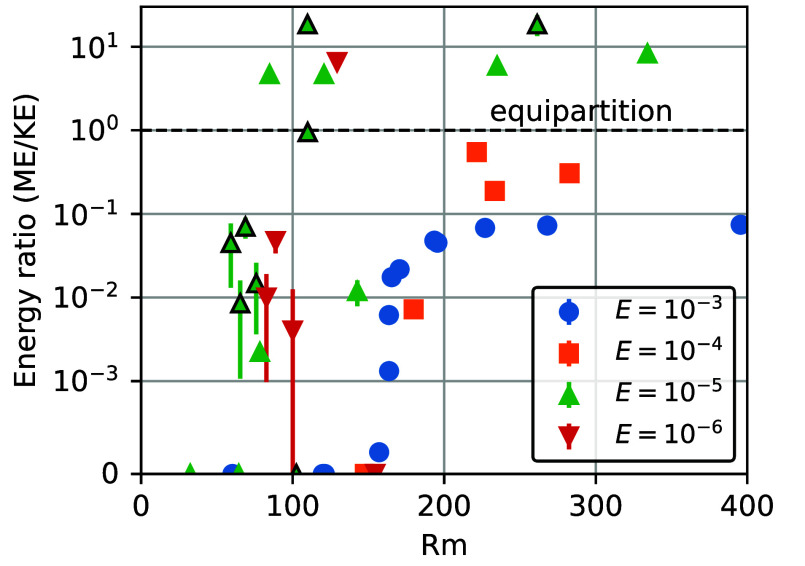
Ratios of magnetic (ME) over kinetic energies (KE) in our dynamo simulations as a function of the magnetic Reynolds number, Rm. Note that the y-axis switches from logarithmic to linear scale below $10^{-3}$. Vertical solid bars denote temporal fluctuations around the mean value. The horizontal dashed line marks equipartition of magnetic and kinetic energies. Symbols outlined in black are for χ=0.8, otherwise χ=0.9.

**Fig. 7.**
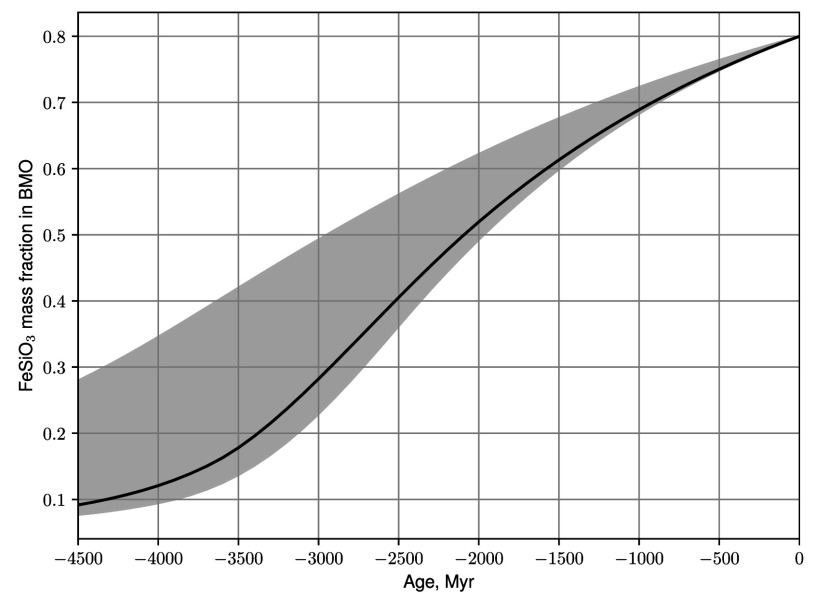
BMO FeO fraction, ξ, as a function of time in the thermal evolution models, made with the same conventions as in prior thermal evolution plots.

Our analyses—based on high-resolution, thin-shell turbulent dynamo models, updated thermal evolution models, and refined convective velocity scalings—suggest that BMO dynamo action may have operated near the threshold of energetic feasibility in early Earth (Fig. 5*A*). Assuming our range of $Q_{0}$ values are accurate and that the higher thin shell $Rm_{\text{crit}}$ and $Rm_{\text{strong}}$ values are robust, long-lived BMO dynamo action requires roughly an order of magnitude higher electrical conductivity values than those proposed in ref. 6. If the BMO electrical conductivity cannot be raised up to the necessary critical values (Fig. 5*B*), then it becomes necessary to identify novel energy sources to drive dynamo action either in the BMO or in the metallic core early on in Earth’s history (e.g., refs. 39, 49, and 64–68). Although our results indicate that sustaining a strong, Earth-like BMO dynamo is energetically challenging, we note that BMO dynamo action could become plausible depending on the behaviors found in more complete treatments of lower mantle phase diagrams, with moderate increases in BMO buoyancy flux or electrical conductivity, with more accurate models of BMO dynamo physics including coupling to a stably stratified metal core, or with changes in the coefficients of rotating scaling predictions. In contrast, BMO dynamos are surely an inevitability in an array of Super Earths and other exoplanetary bodies (e.g., refs. 9 and 69).

## Materials and Methods

### Planetary Dynamo Modeling.

It is effective to work with equations in their nondimensional forms, since the magnetohydrodynamics depends only on a few nondimensional numbers. We adopt the Boussinesq approximation, where the fluid is treated as incompressible, except for the buoyancy term. The length-scale unit is taken to be the gap-width $L=r_{o}-r_{i}$, with $r_{o}$ and $r_{i}$ the outer and inner respective radii of the magma ocean. As time unit, we use the viscous scale: $L^{2}/\nu$, with ν the kinematic viscosity. The nondimensional equations, which govern the evolution of the magnetic b and velocity u fields driven by convection due to a codensity field C (unifying the contributions of temperature and light element composition into a single field), read as (26):

[1] $$\begin{array}{cc} \partial_{t}u+\left(\nabla \times u+2E^{-1}e_{z}\right)\times u & =-\nabla p^{*}+\nabla^{2}u\\ & +\frac{Ra_{F}}{\mathrm{Pr}}\;C+\left(\nabla \times b\right)\times b, \end{array}$$

[2] $$\begin{array}{cc} \partial_{t}b=\nabla \times \left(u\times b\right) & +\frac{1}{\mathrm{Pm}}\nabla^{2}b, \end{array}$$

[3] $$\begin{array}{cc} \partial_{t}C+u.\nabla C & =\frac{1}{\mathrm{Pr}}\nabla^{2}C, \end{array}$$

[4] $$\begin{array}{cc} \nabla u & =0, \end{array}$$

[5] $$\begin{array}{cc} \nabla b & =0. \end{array}$$

Here, the Ekman number $E=\nu /(\Omega L^{2})$ measures the relative effects of viscous to Coriolis forces, with Ω denoting the planetary angular rotation rate. In the early BMO, $E∼10^{-12}$ is expected for a viscosity of 0.1 Pa.s (70), a density of 5,000 kg m^−3^, and a thickness of L=400 km (χ=0.9). For L=800 km and an even lower viscosity (71), the Ekman number could go down to $E∼10^{-14}$, which would tend to make the Rossby number lower still. We study four Ekman numbers: $E=10^{-3}$, $10^{-4}$, $10^{-5}$, and $10^{-6}$. The magnetic Prandtl number is $Pm=\mu_{0}\sigma \nu$, the ratio of the BMO fluid’s kinematic viscosity ν and its magnetic diffusivity ${\left(\mu_{0}\sigma \right)}^{-1}$, with σ the electrical conductivity and $\mu_{0}$ the magnetic permeability of free space. This parameter defines the magnetohydrodynamic properties of the BMO liquid. In silicate melts and liquid metals, $Pm∼10^{-6}$. Dynamo action at such low Pm requires $Re\approx 10^{8}$ to reach Rm=RePm≳100. Such strong turbulence cannot be resolved with current-day computing power (72). With Pm≥0.07 we find self-sustained magnetic fields here for realistic Rm=O(100), the key parameter for dynamo action (73, 74). The Prandtl number Pr=ν/κ, defines the thermomechanical properties of the fluid, where κ is the diffusivity of the codensity field. For pure thermal or pure chemical buoyancy, the diffusivity κ for magma oceans is always smaller than the viscosity, so that Pr≥5, and up to $10^{4}$ for the diffusion of chemical species (70). In this study, we set Pr=5, a compromise between numerical efficiency and Pr≫1. Finally, we employ a buoyancy flux-based Rayleigh number $Ra_{F}=g\beta L^{4}/\nu \kappa$ that controls the vigor of convection (cf., refs. 22 and 23), where β is the imposed codensity gradient at the top of the BMO (parameterizing crystallization on $r_{o}$ yielding denser FeO-enriched fluid). The codensity gradient at the bottom is set to zero (allowing no exchange with the liquid core beneath $r_{i}$). The values of $Ra_{F}$ are chosen to obtain a fairly turbulent flow, as measured by the Reynolds number Re=UL/ν, which has values from 115 (at $E=10^{-3}$) to 1,380 (at $E=10^{-6}$) based on RMS velocity U.

Another implicit parameter is the shell radius ratio, $\chi =r_{i}/r_{o}$, which takes on values of 0.8 and 0.9 here. Spherical dynamos have been widely studied with smaller radius ratios χ≈0.35 motivated by the Earth’s outer core, so that larger χ values represent rather uncharted territory (42, 75, 76). Because of the large χ and Pr values, small-scale convective flows develop that require high angular resolution: we use spherical harmonics up to degree $\ell_{\max}=511$ to 1,999, with hyperdiffusivity for $\ell >0.8\;\ell_{\max}$. For $E\geq 10^{-5}$, the radial grid has $N_{r}=128$ points, $\ell_{\max}=511$. Simulations at $E=10^{-6}$ use higher resolutions (Nr=192, $\ell_{\max}=1,363$), with Nr=256 and $\ell_{\max}=1,999$ for $\left(E,Pm\right)=\left(10^{-6},0.25\right)$.

We impose m-fold azimuthal symmetry to speed up a number of the computations. Each case’s $m_{\mathrm{fold}}$ value is reported in the rightmost column of Table 2. In addition, stress-free conditions are employed at $r_{i}$ and $r_{o}$ to minimize viscous drag effects, since it is not computationally feasible to simulate geophysically realistic, low viscosity fluids ($E≲10^{-12}$) in planetary spherical systems (cf., ref. 78).

**Table 2.** Nondimensional parameters for our thin shell *Pr* = 5 dynamo simulations

Pm	Rm	Re	$Re_{\text{NZ}}$	ME/KE	$f_{\text{dip}}$	$\Lambda_{\text{dip}}$	$m_{\text{fold}}$
$E=10^{-3}$, $r_{i}/r_{o}=0.9$, $Ra_{F}=1.25\times 10^{7}$, $Ra_{F}/Ra_{F}^{\mathrm{onset}}=400$							
1.35	164	121	34.7	0	—	0	4
1.4	164	117	34.7	0.006	0.55	$7.7\times 10^{-3}$	4
1.5	165	110	34.9	0.018	0.55	$1.9\times 10^{-2}$	4
1.6	171	107	35.1	0.022	0.55	$2.1\times 10^{-2}$	4
2	196	97.8	35.2	0.045	0.59	$4.7\times 10^{-2}$	1
2	194	96.8	35.1	0.048	0.59	$4.9\times 10^{-2}$	4
2.5	227	90.8	34.9	0.068	0.58	$6.5\times 10^{-2}$	4
3	268	89.3	34.7	0.073	0.55	$6.0\times 10^{-2}$	4
5	396	79.1	34.8	0.074	0.38	$1.1\times 10^{-2}$	4
10	550	55	35	0.16	0.04	$1.6\times 10^{-5}$	4
20	811	40.6	34.3	0.55	0.18	$4.7\times 10^{-4}$	4
$E=10^{-4}$, $r_{i}/r_{o}=0.9$, $Ra_{F}=2.5\times 10^{8}$, $Ra_{F}/Ra_{F}^{\mathrm{onset}}=370$							
0.3	148	492	76	0	—	0	4
0.5	180	360	80.8	0.007	0.01	$10^{-7}$	4
1	233	233	78.3	0.19	0.64	$2.3\times 10^{-2}$	4
2	221	111	79.1	0.55	0.4	$1.7\times 10^{-2}$	4
3	283	94.2	79	0.31	0.14	$1.3\times 10^{-4}$	4
$E=10^{-5}$, $r_{i}/r_{o}=0.8$, $Ra_{F}=2.5\times 10^{9}$							
0.15	102	683	118	0	—	0	4
0.2	65.4	327	120	0.0085	0.03	$5.9\times 10^{-9}$	4
0.3	76	253	122	0.015	0.07	$2.7\times 10^{-7}$	4
0.375	59.2	158	121	0.045	0.07	$5.0\times 10^{-7}$	4
0.5	68.9	138	121	0.07	0.03	$3.2\times 10^{-7}$	4
0.7	108	155	122	0.92	0.22	$10^{-4}$	1
1	119	119	102	13	0.85	$5.3\times 10^{-2}$	1
2	261	131	92	19	0.85	$9.8\times 10^{-2}$	4
$E=10^{-5}$, $r_{i}/r_{o}=0.9$, $Ra_{F}=2.5\times 10^{9}$, $Ra_{F}/Ra_{F}^{\mathrm{onset}}=172$							
0.1	64.5	645	135	0	—	0	4
0.15	78.4	522	137	0.0022	0.02	$2.9\times 10^{-10}$	1
0.375	143	380	137	0.012	0.04	$3.7\times 10^{-7}$	1
0.7	84.8	121	120	4.7	0.64	$1.4\times 10^{-2}$	1
1	121	121	120	4.7	0.4	$4.6\times 10^{-3}$	1
2	235	117	116	5.9	0.13	$3.2\times 10^{-4}$	1
3	334	111	109	8.3	0.41	$8.5\times 10^{-3}$	4
$E=10^{-6}$, $r_{i}/r_{o}=0.9$, $Ra_{F}=10^{11}$, $Ra_{F}/Ra_{F}^{\mathrm{onset}}=322$							
0.05	≈140	≈2,800	≈382	0	–	0	8
0.07	100	1,430	387	0.004	0.08	$10^{-9}$	8
0.1	82.7	827	387	0.01	0.68	$1.2\times 10^{-6}$	8
0.15	88.8	592	383	0.047	0.24	$1.5\times 10^{-6}$	8
0.25	129	517	295	6.5	0.9	$3.9\times 10^{-2}$	8

$Ra_{F}^{\mathrm{onset}}$ is the $Ra_{F}$ value at the onset of nonmagnetic, rotating convection, measured at $E=10^{-5}$ (to within 4%) and extrapolated using $Ra_{F}^{\mathrm{onset}}\propto E^{-4/3}$. The Reynolds number Re ($Re_{\mathrm{NZ}}$) is the ratio of inertial and viscous forces using the RMS (nonzonal) velocity. The volume-integrated ratio of the magnetic and kinetic energies is ME/KE. The fraction of dipolar magnetic field at the surface of the BMO is $f_{\text{dip}}$, and the Elsasser number $\Lambda_{\mathrm{dip}}=E\;Pm\;B_{\mathrm{dip}}^{2}$ measures the square of the magnetic dipole amplitude $B_{\mathrm{dip}}$ on $r=r_{o}$. More simulation output quantities are available at https://doi.org/10.6084/m9.figshare.28551332.v2 (77).

We solve Eqs. **1**–**4** using the freely available XSHELLS code (79) on a cluster of GPUs that are faster and more energy efficient than traditional CPUs. Input and output parameter values for all simulations are shown in Table 2 and summarized in Fig. 6.

### Thermal Evolution Model.

The thermal evolution model closely follows the one originally proposed by Labrosse et al. (1) and used by Ziegler and Stegman (5) and Stixrude et al. (6) with a few modifications, most notably in the use of more realistic phase diagrams and the inclusion of the compositional energy term in the energy balance. We differ from refs. 5 and 6, however, in the predictions for the buoyancy flux available to drive the dynamo. The energy balance states that the heat flow entering the solid mantle is balanced by the sum of the secular cooling of the BMO and core, the latter being equal to the CMB heat flow in our simple model, the radiogenic heating in the BMO, the latent heat of BMO crystallization and the compositional energy from FeO fractionation:

$$\begin{array}{cc} 4\pi r_{o}^{2}k\frac{T_{L}-T_{m}}{\delta } & =-\left(M_{\mathrm{BMO}}C_{m}+M_{c}C_{c}\right)\frac{\partial T_{L}}{\partial t}\\ & +H\left(t\right)-4\pi r_{o}^{2}\rho \Delta ST_{L}\frac{dr_{o}}{dt}+E_{\mathrm{FeO}}, \end{array}$$

with $T_{L}$ and $T_{m}$ the liquidus and bulk mantle temperature; δ the thickness of the boundary layer at the bottom of the solid mantle; $M_{\mathrm{BMO}}=\left(4/3\right)\pi \left(r_{o}^{3}-r_{i}^{3}\right)\rho$ the mass of the BMO; $\rho =5.5\;\text{kg}\;{\text{m}}^{-3}$ its density; $M_{c}=2\times 10^{24}\;\text{kg}$ the mass of the core; $C_{m}=1\times 10^{3}\text{J}\;{\text{K}}^{-1}\;{\text{kg}}^{-1}$ and $C_{c}=750\;\text{J}{\text{K}}^{-1}\;{\text{kg}}^{-1}$ the specific heat of mantle and core material, respectively; H(t) the total radiogenic heating rate of the BMO; and $\Delta S=652\;\text{J}\;{\text{K}}^{-1}\;{\text{kg}}^{-1}$ (6) the entropy of melting. The compositional energy term is proportional to the rate of change of the FeO mass fraction ξ (43),

$$\begin{array}{c} E_{\mathrm{FeO}}=-\rho \alpha_{\xi }g\left[r_{o}V_{\mathrm{BMO}}-\pi \left(r_{o}^{4}-r_{i}^{4}\right)\right]\frac{\text{d}\xi }{\text{d}t}, \end{array}$$

where $V_{\mathrm{BMO}}=M_{\mathrm{BMO}}/\rho$ is the volume of the BMO and $\alpha_{\xi }=-\rho^{-1}\frac{\partial \rho }{\partial \xi }=-1$ is the chemical (FeO) expansion coefficient.

The FeO mass fraction ξ in the BMO increases by fractional crystallization following its conservation equation

$$\begin{array}{c} \frac{\text{d}\xi }{\text{d}t}=-\frac{3r_{o}^{3}\Delta \xi }{r_{o}^{3}-r_{i}^{3}}\frac{\text{d}r_{o}}{\text{d}t}, \end{array}$$

with Δξ the FeO mass fraction difference between the BMO and the solid mantle. Instead of using a constant value for this parameter as in ref. 1, we compute it using the same phase diagram as used by Boukare et al. (2), which considers the temperatures of the liquidus $T_{L}$ and the solidus $T_{S}$ to vary as

$$\begin{array}{cc} T_{L} & =T_{\mathrm{Fe}}+\left(T_{\mathrm{Mg}}-T_{\mathrm{Fe}}\right)\left(1-\xi^{1.7}\right),\\ T_{S} & =T_{\mathrm{Fe}}+\left(T_{\mathrm{Mg}}-T_{\mathrm{Fe}}\right)\xi^{1.1}, \end{array}$$

taking $T_{\mathrm{Mg}}=5,000\;\text{K}$ and $T_{\mathrm{Fe}}=3,400\;\text{K}$ as the melting temperatures assumed for the MgO-rich and FeO-rich endmembers, respectively. An alternative phase diagram based on the melting model of Katz (80) is also tested (*SI Appendix*) as it gives a larger degree of FeO fractionation. Reducing the phase diagram to a binary system is expected to be valid for the early times of BMO crystallization (81), which is the main concern of this paper. This assumption would certainly break down when getting close to complete freezing, where various other phases become important. This means that the precise timing for the later evolution of the BMO cannot be considered well resolved in our model (7).

The equations above can be combined to obtain a set of two coupled ordinary differential equations describing the temporal evolution of the BMO outer radius $r_{o}$ and its FeO mass fraction ξ, which are solved using a python script that is available on SoftwareHeritage: ThermEvolBMO. Note that more complete sets of equations for the evolution of the BMO have been developed by Blanc et al. (7) and Lherm et al. (8). Further, Labrosse et al. (43) take into account the possibility of dynamically induced melting and freezing across the phase change as a modified mechanical boundary condition (82). The main dynamical effect of this phase change boundary condition is that it facilitates convection in the solid by decreasing the critical Rayleigh number for the onset of convection and increases the efficiency of heat transfer by roughly a factor of 2 (83). This effect is accounted for by varying the current value $Q_{0}$ of heat flow at $r_{o}$. This value is used to compute the thickness of the boundary layer, δ, which is assumed constant, as in the original BMO paper (1). Beyond an Occam’s razor argument, this assumption is justified by the idea that the heat flux at the bottom of the solid mantle is dominated by the arrival of cold plates that spread to form the boundary layer (84). Using a constant boundary layer thickness leads to a factor ∼3 variation of the BMO heat flow with time (Fig. 3). Alternatively, a boundary layer scaling law could be used as in ref. 8, which could provide a higher initial value of the BMO heat flow, leading to a faster cooling and possibly a short-lived dynamo. Such classical scaling laws, however, do not accurately represent the dynamics associated with a phase change boundary condition (43, 82, 83). Our choice of a constant δ could be seen as the result of continuous action of plate tectonics through time, which is a controversial topic, but including other options falls beyond the scope of this work.

Using the updated thermal evolution model, we compute the buoyancy flux available to drive BMO convection and evaluate its potential for generating dynamo action therein. The thermal buoyancy comes from the difference between the total heat flux out of the BMO and the latent heat,

$$\begin{array}{c} q^{\prime }=k\frac{T_{L}-T_{m}}{\delta }+\rho \Delta ST_{L}\frac{\text{d}r_{o}}{\text{d}t}, \end{array}$$

since the latent heat produced at the top of the BMO acts to suppress BMO convection. With the latent heat production dominant in the early evolution of the model, it acts to subtract heat from the total outward heat flux and lowers the expected convective power compared to what was considered in previous studies (5, 6). However, we also consider an additional source of buoyancy: the compositional buoyancy resulting from the release of FeO during fractional crystallization at the top of the BMO, which is proportional to the BMO’s crystallization rate. The total buoyancy flux, $I_{b}$, is then

$$\begin{array}{c} I_{b}=\frac{\alpha gq^{\prime }}{\rho C_{m}}-\Delta \xi g\frac{\text{d}r_{o}}{\text{d}t}. \end{array}$$

### Convective Velocity Estimates.

The buoyancy flux $I_{b}$ is then used to calculate the three convection velocities considered here, in m/s, following (e.g., refs. 14, 16, 20, 21, and 52). The nonrotating free-fall (or mixing length) velocity is estimated via

$$\begin{array}{c} U_{\mathrm{FF}}={\left(I_{b}\;L\right)}^{1/3}. \end{array}$$

The rapidly rotating, thermal wind scaling velocity is calculated as

$$\begin{array}{c} U_{\mathrm{ThW}}={\left(I_{b}^{2}L/\left(2\Omega \right)\right)}^{1/5}. \end{array}$$

The magnetostrophic velocity is estimated from

$$\begin{array}{c} U_{\mathrm{MAC}}={\left(I_{b}/\left(2\Omega \right)\right)}^{1/2}. \end{array}$$

Note that we have assumed coefficients all equal to unity in these velocity scalings. Furthermore, these scalings ignore convectively driven zonal flows, which are present in our thin spherical shells and can contribute to dynamo action. The $U_{\mathrm{ThW}}$ scaling also ignores the effects of magnetic braking that will tend to lessen the flow speeds, especially when $Rm\geq Rm_{\text{strong}}$ (31, 85). These velocity estimates make it possible to estimate i) the ratio of inertial and viscous accelerations in the fluid via the Reynolds number, Re=UL/ν; ii) the Rossby number, Ro=U/(2ΩL), which approximates the ratio of inertial and Coriolis accelerations, and iii) the magnetic Reynolds number, $Rm=\mu_{o}\sigma UL$, which estimates the ratio of magnetic induction versus dissipation.

## Supplementary Material

Appendix 01 (PDF)

## Data Availability

The XSHELLS dynamo code used is freely available at https://nschaeff.bitbucket.io/xshells/ (79). Numerical simulation outputs data have been deposited in Figshare (https://doi.org/10.6084/m9.figshare.28551332.v2) (77).
