# Supplementary material for: Energetically expensive dynamo action in Earth’s basal magma ocean
Source: Proc Natl Acad Sci U S A. 2025 Nov 3;122(45):e2507575122. doi: 10.1073/pnas.2507575122 (PMC12625979; doi:10.1073/pnas.2507575122)
Supplement: Supplementary file 1 — Appendix 01 (PDF) [file pnas.2507575122.sapp.pdf]

Supporting Information for

Energetically Expensive Dynamo Action in Earth's Basal Magma Ocean

N. Schaeffer, S. Labrosse, J.M. Aurnou

Corresponding Author name.
E-mail: aurnou@ucla.edu

This PDF file includes:

Figs. S1 to S5
SI References

Overview. This supplement shows the free-fall scaling predictions solely for comparison to other studies, noting that we have shown that they do not accurately describe flows in Earth's basal magma ocean (BMO). Further, an alternate set of thermal evolution models is calculated based on a different FeO phase diagram that is based on the work of (1). The change in phase diagram does not alter the conclusions of the main text.

Evolution of the magnetic Reynolds number including the free-fall scaling law for non-rotating convection

The "free-fall" (FF) velocity scaling cannot be applied to the BMO. As shown on Fig. 5a, the Rossby number, Ro, of the BMO is expected to be much lower than 1 for its whole history. When $Ro \ll 1$, rotational effects dominate over fluid inertia, thus, disallowing non-rotating, inertially dominated FF dynamics (2).

The FF scaling has, however, been used by (3) to investigate the possibility of dynamo action in the BMO to explain Earth's early magnetic field. Although we excluded the non-physical FF calculation from Figure 5 of the main text, it is included here in Fig. S1 for completeness. It can be seen that the FF scaling provides values of Rm that are larger than the thermal wind and MAC balance scalings that we consider acceptable for the BMO. The FF result is similar to the one obtained by (3). This, combined with the lower $Rm_{crit}(\chi = 0.35) \approx 40$ value of the critical magnetic Reynolds number used in previous studies, allows them to argue for BMO dynamo action as the origin of the early geomagnetic field.

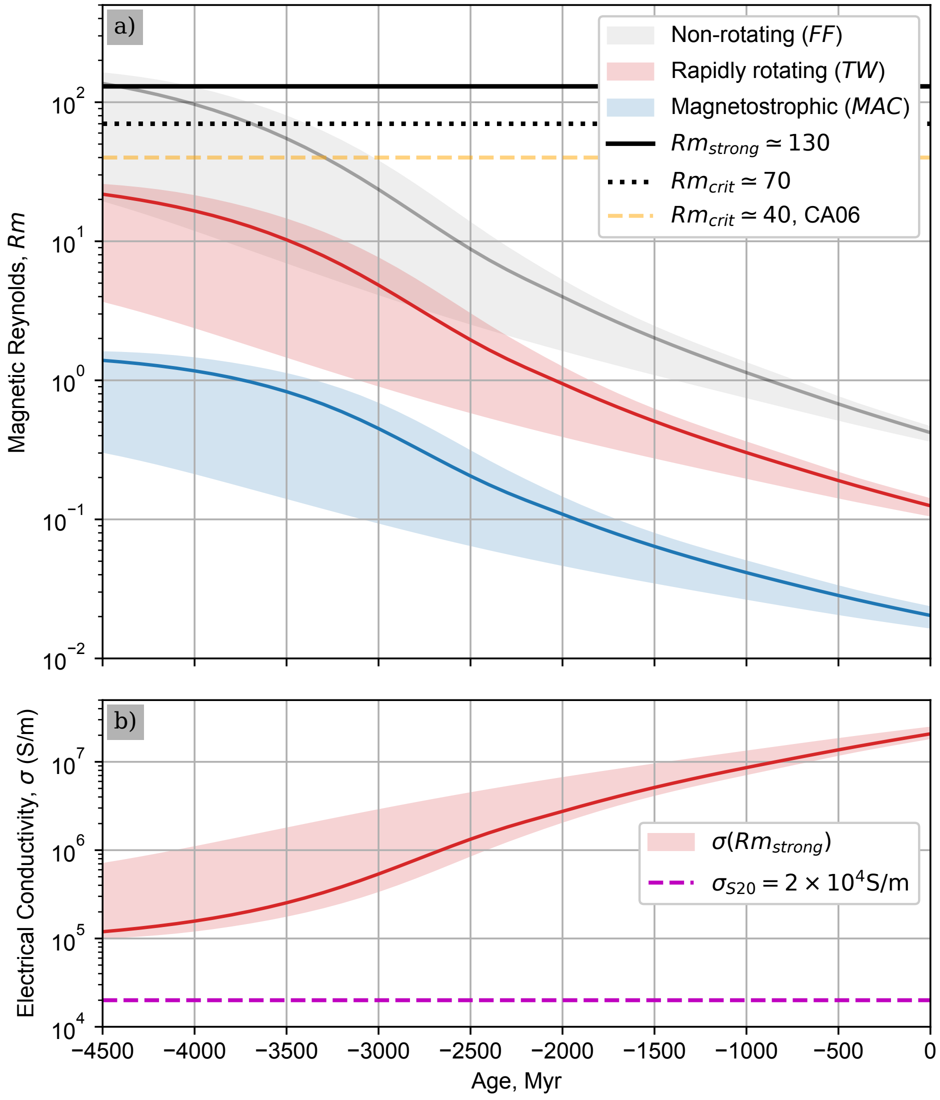

Fig. S1. Same as Fig. 5 of the main paper, with the addition of the "free-fall" (FF) scaling for the magnetic Reynolds number.

Alternative phase diagram

The phase diagram used to compute the figures in the main text, as explained in the Methods section, is the one used by (4). It has been designed to fit the experimental results at the pressure of the lower mantle. However, it has a rather large partition coefficient, increasing from 0.15 to 0.8 over the course of the evolution. In order to test the sensitivity of the phase diagram, we have implemented here another formulation derived from the one proposed by (1) for olivine, adapted for the numerical values relevant to Bridgmanite in the lowermost mantle. This phase diagram features a lower partition coefficient, therefore providing a larger Fe release at the top of the BMO upon fractional crystallization. Here, we provide the equations solved using this formulation. Both formulations are implemented in the python script `ThermEvolBMO.py` available on SoftwareHeritage: ThermEvolBMO.

The formulation is taken from Chapter 10 of (1). We assume a solid-solution between two end-members, $MgSiO_3$ and $FeSiO_3$, with melting temperatures T_{Mg} and T_{Fe}, respectively. These are associated to latent heats L_x and modified gas constants $R_x = R/M_x$ for each end-member x, where M_x is the molar mass of component x. The partition coefficient of component x between the liquid and the solid is the ratio of the mass fraction of the x end-member in the solid, ξ_x^s, to that in the liquid, ξ_x^l,

$$K_x(T) = \frac{\xi_x^s}{\xi_x^l} = \exp\left[\frac{L_x}{R_x}\left(\frac{1}{T} - \frac{1}{T_x}\right)\right]. \quad [1]$$

Since we consider only two components, $\xi_{Fe} + \xi_{Mg} = 1$ for both phases and, in following, we note $\xi \equiv \xi_{Fe}$ and use $1 - \xi$ instead of ξ_{Mg}. eq:K is complemented by the lever rule which leads to equation 10.20 of (1):

$$\frac{\bar{\xi}(1 - K_{Fe})}{f + (1 - f)K_{Fe}} + \frac{(1 - \bar{\xi})(1 - K_{Mg})}{f + (1 - f)K_{Mg}} = 0. \quad [2]$$

In the expression above, f is the melt fraction and the average mass fraction is

$$\bar{\xi} = f\xi^l + (1 - f)\xi^s = \xi^l\left[f + (1 - f)K_{Fe}\right]. \quad [3]$$

Given the relevant quantities for the end-members, these equations can be used to get the mass fractions of each component in each phase for any temperature T. In particular, the solidus composition is given by setting $f = 0$ and the liquidus by setting $f = 1$. Solving for ξ in the latter case gives mass fraction of the Fe end-member at the liquidus as

$$\xi_{liq}(T) = \frac{K_{Mg}(T) - 1}{K_{Mg}(T) - K_{Fe}(T)}. \quad [4]$$

The mass fraction at the solidus is then $\xi_{sol} = K_{Fe}\xi_{liq}$.

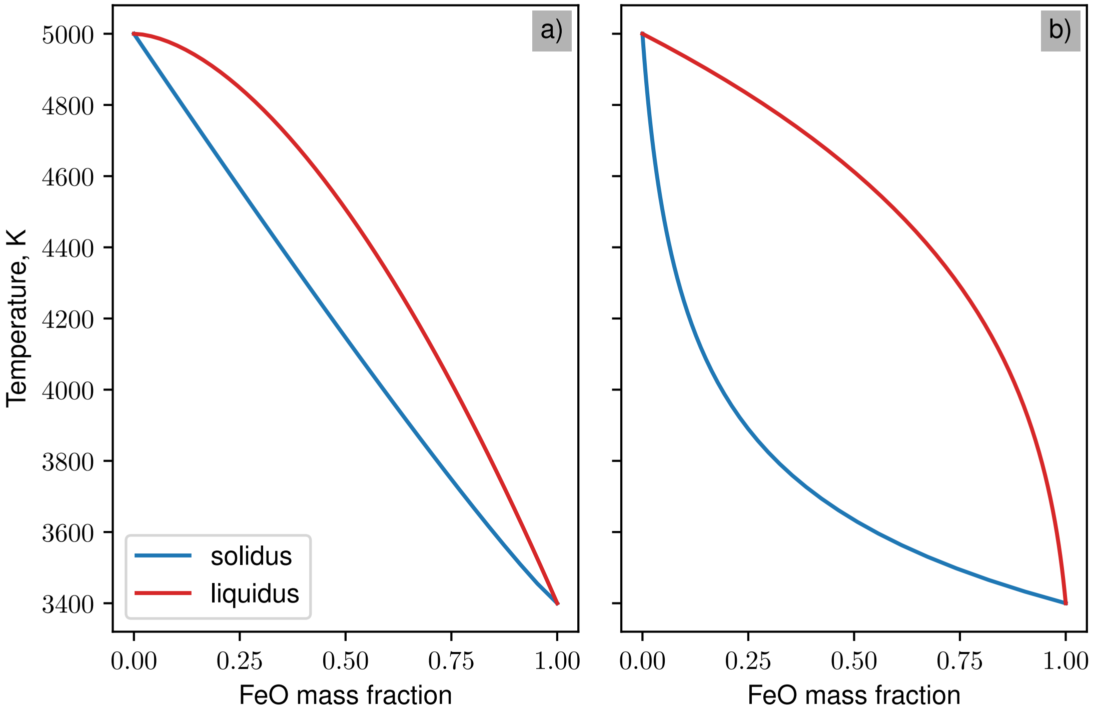

Fig. S2. Phase diagrams used in the model. a) phase diagram used in the main text, based on the formulation by (4). b) phase diagram based on the formulation by (1).

For the thermal evolution model, we need to relate variations of the liquidus temperature to that of the liquidus composition, which can be done by differentiating eq:xi-liquidus to get

$$T_L\xi_{liq} = -\frac{T_L^2(K_{Mg}-K_{Fe})}{\xi_{liq}\frac{L_{Fe}}{R_{Fe}}K_{Fe}+(1-\xi_{liq})\frac{L_{Mg}}{R_{Mg}}K_{Mg}}. \qquad [5]$$

In this formulation, instead of using ξ and r_o as control parameters to follow the thermal evolution, we use T_L and r_o. To that end, the equation for ξ/t in the methods section is replaced by

$$T_Lt = -\frac{3r_o^3\Delta\xi(T_L)}{r_o^3-r_i^3}T_L\xi_{liq}r_ot. \qquad [6]$$

Switching formulation in the `python` script amounts to choosing either `'xi'` or `'TL'` for the variable `evolpar`, respectively for an evolution parameterized by ξ or T_L. Again, the script ThermEvolBMO really is freely available on SoftwareHeritage.

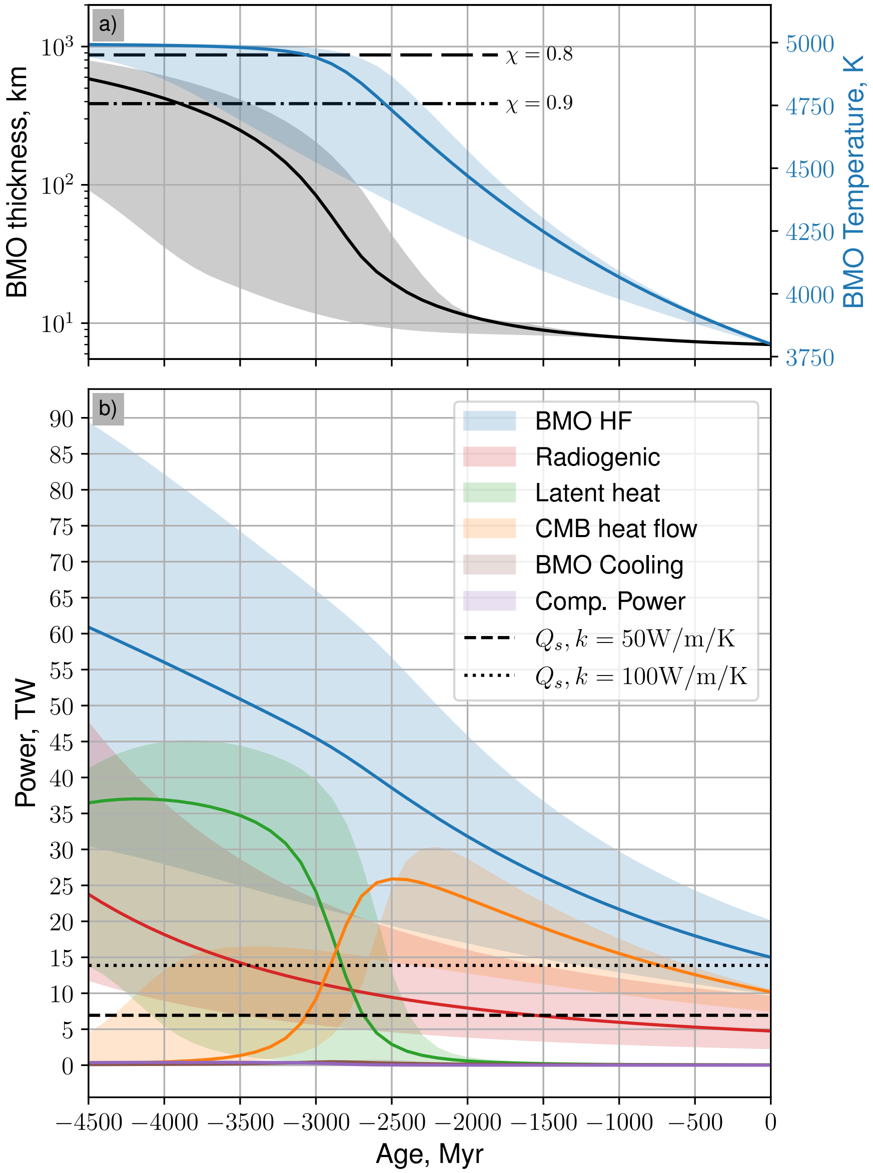

Fig. S3. Thermal evolution modeling results based on the phase diagram formulation of (1) for current-day heat flow values $Q_0 = 10; 15; 20$. The central value for each quantity is plotted as solid line, the shaded area giving the range between the two extremum scenarios. a) BMO thickness $L = r_o - r_i$ (grey and black, left axis) and bulk temperature (blue, right axis). b) Curves showing the heat flux leaving the BMO into the overlying mantle (blue); radiogenic BMO heating (red); latent heat of crystallization at r_o (green); CMB heat flow (orange); BMO secular cooling (brown); BMO compositional power (purple), all in units of TW. The two horizontal dashed and dotted lines give isentropic CMB heat flow at the top of the core for a thermal conductivity $k_c 50; 100$, respectively.

This formulation has been used with the same parameters as the one used for the phase diagram of (4). In the absence of any constraint on the latent for the end-members, we used the same value for both end-members, obtained from the entropy of melting $\Delta S = 652$ (3) as $L_x = T_x \Delta S$. Allowing for changes in this parameter with composition would be straightforward but our tests show that these affect only mildly the results.

Fig. S2 shows the phase diagrams resulting from the two formulations. The phase diagram using the formulation by (1) is open more widely, which implies a lower partition coefficient, varying from 0.04 to 0.3 during the course of the evolution. This leads to more fractionation of FeO between the solid and the liquid. Implications for the energy balance and the evolution of the BMO radius are shown in Fig. S3, counterpart to Figure 3 in the main text. The most important implication is a significantly larger initial BMO thickness compared to that obtained with the formulation of (4), around 600 for the central value compared to 400. Consequently, the various energy terms are also larger. The evolution of the various terms are similar using both formulations, the one using the formulation by (1) having a more abrupt transition between the early regime where latent heat dominates over CMB heat flow to the later reversed situation. This can be explained by a larger variation of the partitioning behaviour with time.

Fig. S4 is the counterpart to Fig. 4 in the main text and shows results that are of the same order of magnitude. The scaling exponents that give the estimates of the flow velocity as function of the energy terms are all smaller than 1/2 which explains why the roughly 50 increase in heat flow and other power terms compared to the large K formulation makes little difference in the Rossby and Reynolds number estimates. As for the power balance, the time evolution shows a more abrupt transition. The small values obtained for the Rossby number reinforce the prediction that the dynamics of the BMO is largely controlled by the Coriolis force. Thus, non-rotating FF velocities are still disallowed in this alternative formulation.

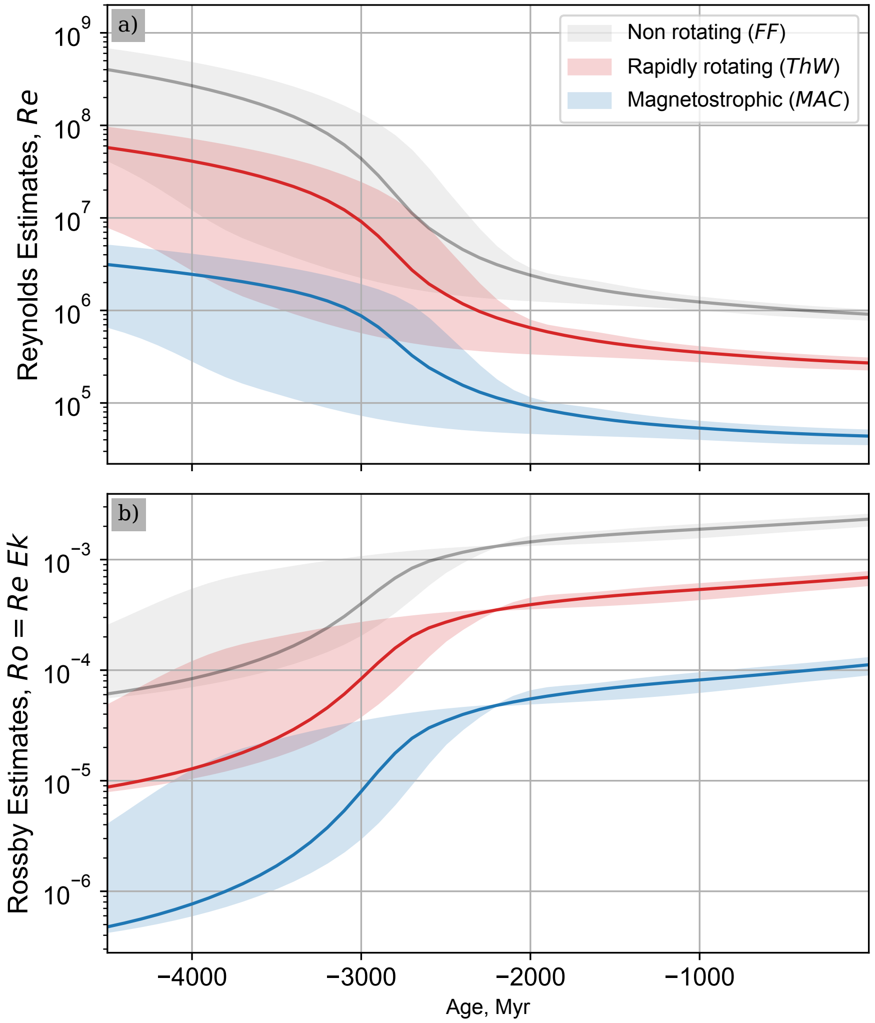

Fig. S4. Nondimensional BMO convective velocity estimates based on the power outputs from the (1)-based thermal evolution model for current-day heat flow values $Q_0 = 10; 15; 20$. a) Reynolds numbers, Re, estimate the ratio of inertial to viscous forces, whereas b) Rossby numbers, $Ro = ReE$, measure the ratio of inertial to Coriolis forces. The grey curve corresponds to the non-rotating free-fall (FF) velocity scaling. The red curve marks the rapidly rotating thermal wind (ThW) scaling. The blue curve denotes the magnetostrophic (MAC) scaling.

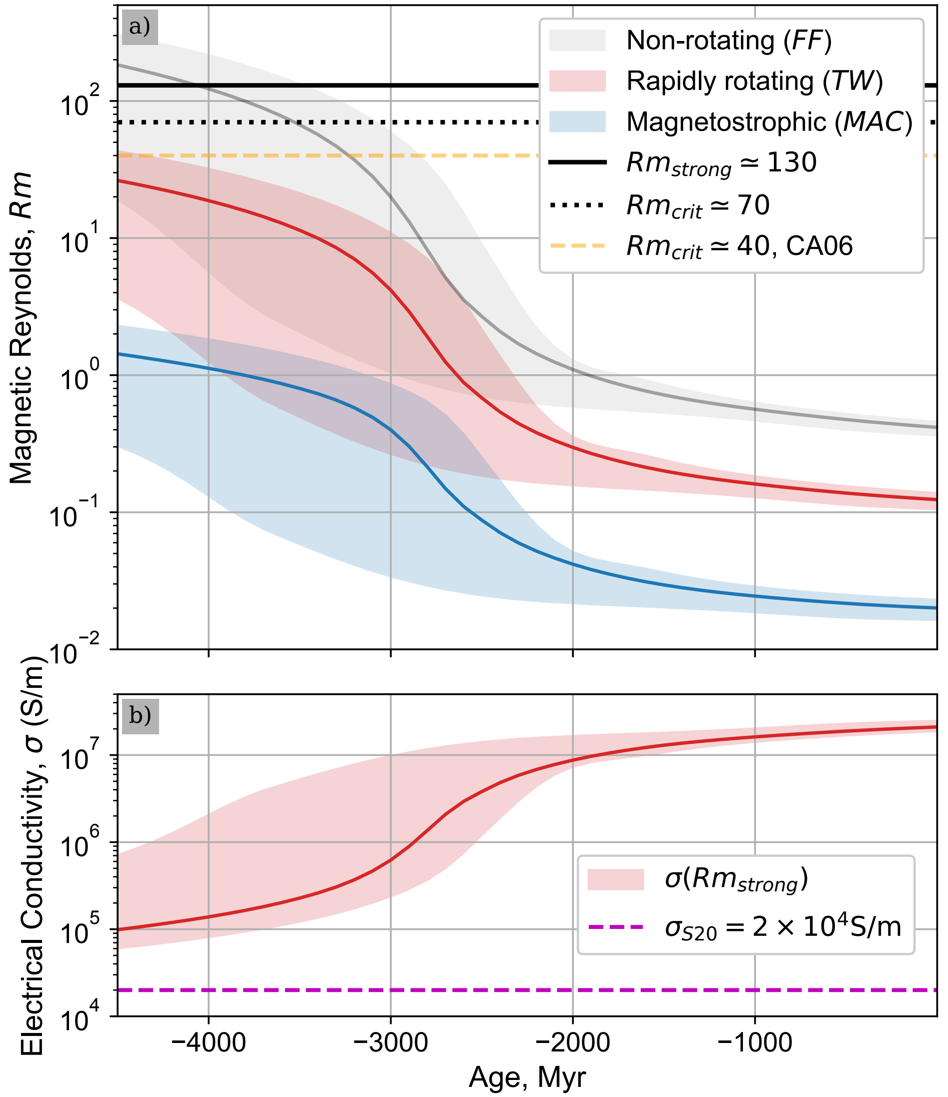

Fig. S5. a) Magnetic Reynolds number estimates as a function of Earth's age for the different velocity scalings considered here. The critical value, $Rm_{\rm crit}$, above which dynamo action occurs is estimated to be 70 in our thin shell BMO dynamo models (black dashed line) and 40 in thick shell Earth's outer core models (orange dashed line). b) Estimated values of BMO electrical conductivity, $\sigma \approx Rm_{\rm strong}/(\mu_o U_{ThW} L)$, necessary to drive strong field dynamo action for thermal wind convection velocities for thermal evolution paths with the range of heat flow considered here. For comparison, the magenta dot-dashed line denotes $\sigma_{S20} = 2 \times 10^4$ S/m from (3).

Finally, Fig. S5 is the counterpart to Fig. 5 of the main text. The magnetic Reynolds numbers that result from the two relevant velocity scaling laws, ThW and MAC, are slightly larger than those obtained using the phase diagram of (4), with a maximum Rm value using the ThW scaling that grazes the critical value for a thick shell dynamo, $Rm_{crit}(\chi = 0.35) \approx 40$. Therefore, even for a larger partitioning between the solid and the liquid, we find that maintaining and sustaining the early magnetic field with a BMO-based convective dynamo is energetically challenging.

References

1. RF Katz, *The dynamics of partially molten rock.* (Princeton University Press, Princeton), (2022).
2. JM Aurnou, S Horn, K Julien, Connections between nonrotating, slowly rotating, and rapidly rotating turbulent convection transport scalings. *Phys. Rev. Res.* **2**, 043115 (2020).
3. L Stixrude, R Scipioni, MP Desjarlais, A silicate dynamo in the early earth. *Nat. communications* **11**, 1–5 (2020).
4. CÉ Boukaré, J Badro, H Samuel, Solidification of Earth's mantle led inevitably to a basal magma ocean. *Nature* **640**, 114–119 (2025).
